# Anti-*Agrobacterium tumefactions* sesquiterpene derivatives from the marine-derived fungus *Trichoderma effusum*

**DOI:** 10.3389/fmicb.2024.1446283

**Published:** 2024-08-02

**Authors:** Yunfeng Liu, Lu Qi, Minghui Xu, Wanyun Li, Na Liu, Xueli He, Yuxing Zhang

**Affiliations:** ^1^College of Horticulture, Hebei Agricultural University, Baoding, China; ^2^College of Life Sciences, Hebei University, Baoding, China; ^3^College of Pharmaceutical Sciences, Hebei University, Baoding, China

**Keywords:** marine-derived fungus, *Trichoderma effusum*, sesquiterpene, *Agrobacterium tumefaciens*, bioactivity

## Abstract

*Agrobacterium tumefaciens* can harm various fruit trees, leading to significant economic losses in agricultural production. It is urgent to develop new pesticides to effectively treat this bacterial disease. In this study, four new sesquiterpene derivatives, trichoderenes A−D (**1**–**4**), along with six known compounds (**5**–**10**), were obtained from the marine-derived fungus *Trichoderma effusum*. The structures of **1**–**4** were elucidated by extensive spectroscopic analyses, and the calculated ECD, ORD, and NMR methods. Structurally, the hydrogen bond formed between the 1-OH group and the methoxy group enabled **1** to adopt a structure resembling that of resorcylic acid lactones, thereby producing the ECD cotton effect. Compound **3** represents the first example of C12 nor-sesquiterpene skeleton. Compounds **1**–**10** were tested for their antimicrobial activity against *A. tumefactions*. Among them, compounds **1**–**3** and **8**–**10** exhibited inhibitory activity against *A. tumefactions* with MIC values of 3.1, 12.5, 12.5, 6.2, 25.0, and 12.5 μg/mL, respectively.

## Graphical abstract

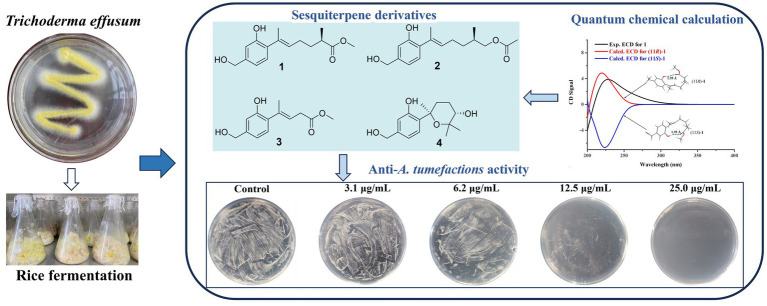

## 1 Introduction

*Agrobacterium tumefactions* is a prevalent gram-negative bacterium found in soil, which exhibits a remarkable ability to infect the wounded sites of various fruit trees, including pear, apple, peach, kiwi, and cherry trees, under natural conditions (Yu et al., 2021; Hang et al., 2022). This infection has the propensity to induce the development of crown gall disease, a pathological condition that primarily affects the root and stem of plants (Ahmed et al., 2022). The disease is characterized by the emergence of small, round, light-yellow tumors on the infected sites, with diameters ranging from a few millimeters to several centimeters. As the disease progresses, these tumors enlarge and assume irregular shapes, ultimately leading to a substantial reduction in crop yield and, in severe cases, even plant death (Jailani et al., 2022). Currently, there is no highly effective method for treating crown gall disease, and the commonly used chemical agents for prevention can cause serious environmental pollution problems (Torres et al., 2022). It is urgent to develop new natural and green pesticides with a high degree of effectiveness and low environmental impact.

*Trichoderma* species are dominant fungal communities in various soil ecosystems across all climatic zones, serving as a crucial component of the soil microecological flora and possessing the ability to colonize plant roots (Zin and Badaluddin, 2020). Recent research has revealed that *Trichoderma* not only exhibits remarkable adaptability but also effectively controls various plant diseases and pests (Ferreira and Matías, 2021). On the one hand, its rapid growth and strong vitality enable it to swiftly occupy growth spaces, absorb necessary nutrients, and weaken and eliminate other pathogens in the same environment (Harman et al., 2012). On the other hand, *Trichoderma* inhibits the growth, reproduction, and infection of pathogenic bacteria through the production of small-molecule antibiotics, large-molecule antibacterial proteins, or cell wall-degrading enzymes (Tyśkiewicz et al., 2022). Technical measures employing *Trichoderma* in the prevention and control of fruit and vegetable diseases have garnered widespread attention in the field of biological control both domestically and internationally (Cai and Druzhinina, 2021). Currently, over 250 commercial formulations containing *Trichoderma* have been developed globally, achieving remarkable control effects in different countries and regions (Mukhopadhyay and Deepak, 2020). The research on biological control and mechanisms of *Trichoderma* is of significant importance for promoting biological control and reducing the use of chemical pesticides.

**Figure 1:**
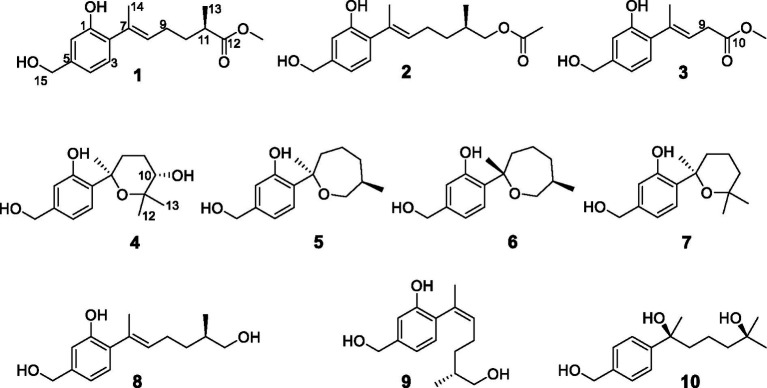
Structures of the compounds **1**–**10**.

As part of our ongoing search for antibacterial natural products from marine-derived fungi (Li et al., 2022; Cao et al., 2023), the strain *Trichoderma effusum* attracted our attention because the EtOAc extract of the culture showed anti-*A. tumefactions* activity. The bioassay and HPLC guided separation of the EtOAc extract led to the isolation of four new sesquiterpene derivatives, named trichoderenes A−D (**1**–**4**), together with six known compounds (**5**–**10**) (Figure 1). Subsequently, anti-*A. tumefactions* activities of these compounds (**1**–**10**) were performed to evaluate the development value of these compounds. Herein, we report the details of the isolation, structure elucidation, and bioactivities of them.

## 2 Results and discussion

**Figure 2:**
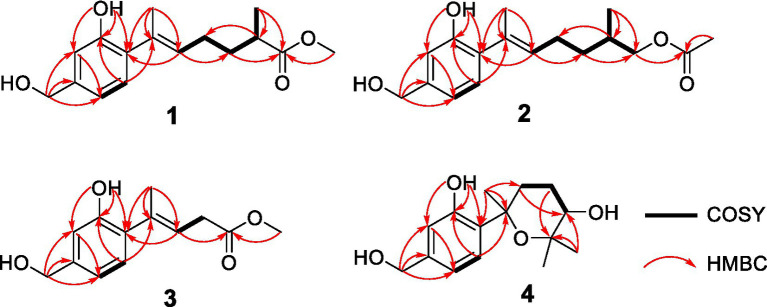
Key HMBC and COSY of compounds **1**–**4**.

Trichoderene A (**1**) was obtained as pale yellow oil with the molecular formula of C_16_H_22_O_4_ based on its HRESIMS (*m/z* 301.1404 [M + Na]^+^, calcd. for C_16_H_22_ NaO_4_^+^ 301.1410), suggesting six degrees of unsaturation. In the ^1^H NMR spectrum (Table 1), three characteristic aromatic proton signals [7.05, d (7.2), 6.93, d (1.2), and 6.88, d (7.2, 1.2)], an olefinic methylene signal [5.51, tb (7.2, 1.2)], an oxygen-linking methylene signal [4.63, d (5.4)], and two methyl proton signals [1.96, s; 1.20, d (7.2)] revealed a bisabolene-type sesquiterpene skeleton (Shu et al., 2021) for **1**. Moreover, in the ^13^C NMR spectrum of **1** (Table 2), 17 related carbon atoms, which could be assigned to three methyl groups including one methoxy group, three methylenes, five methines, and five quaternary carbons including a carbonyl group, were consistent with the ^1^H NMR of **1**. Compared with the reported NMR data of anhydrowuruterpol B (**8**) isolated from the fungus *Penicillium* sp. FH-A 6260 (Henne et al., 1993), it was suggested that compound **1** shares the same bisabolene-type nucleus as compound **8**. The main difference was that the methyl ester group in **1** [*δ*_H_ 3.69, s, -OC *H* _3_; *δ*_C_ 177.3 C-12 and 51.8 -O *C* H_3_] was instead of primary alcohol group in **8**. This deduction was confirmed by the key HMBC correlations from H-10, H-13, and -OC *H* _3_ to C-12 (Figure 2). Based on the above analysis, the planar structure of **1** was established.

**Table 1:** ^1^H NMR Data (*δ*) of **1**–**4** (600 MHz, CDCl_3_, *δ* in ppm, *J* in Hz).

Position	1	2	3	4
3	7.05, d (7.2)	7.08, d (7.8)	7.00, d (7.8)	7.03, d (7.8)
4	6.88, d (7.2, 1.2)	6.90, dd (7.8, 1.2)	6.93, dd (7.8, 1.2)	6.83, dd (7.8, 1.8)
6	6.93, d (1.2)	6.94, d (1.2)	6.95, d (1.2)	6.86, d (1.8)
8	5.51, tb (7.2, 1.2)	5.55, tb (7.2, 1.2)	5.81, tb (7.2, 1.2)	2.29, m
				2.07, m
9	2.24, m	2.29, m	2.90, d (7.2)	2.04, m
		2.22, m		1.81, m
10	1.84, m	1.56, m	-	3.48, m
	1.59, m	1.34, m		
11	2.53, m	1.86, m	-	-
12	-	4.01, dd (10.8, 6.6)	-	1.31, s
	-	3.91, dd (10.8, 6.6)		
13	1.20, d (7.2)	1.00, d (7.2)	-	1.01, s
14	1.96, s	2.00, s	2.04, s	1.53, s
15	4.63, d (5.4)	4.65, d (5.4)	4.65, s	4.63, s
-OCH3	3.69, s	-	3.68, s	-
-OAc	-	2.08, s	-	-
1-OH	5.72, s	5.60, s	5.82, s	8.96, s

**Table 2:** ^13^C NMR Data (*δ*) of **1**–**4** (150 MHz, CDCl_3_, *δ* in ppm).

Position	1	2	3	4
1	152.4, C	152.3, C	152.4, C	157.0, C
2	130.6, C	130.5, C	126.4, C	130.2, C
3	128.7, CH	128.7, CH	128.9, CH	124.6, CH
4	118.8, CH	118.8, CH	119.3, CH	118.2, CH
5	141.4, C	141.4, C	142.2, C	142.1, C
6	114.2, CH	114.0, CH	114.8, CH	116.2, CH
7	133.0, C	132.4, C	136.8, C	78.0, C
8	130.7, CH	131.4, CH	122.3, CH	28.1, CH_2_
9	26.4, CH_2_	25.9, CH_2_	34.8, CH_2_	24.3, CH_2_
10	33.4, CH_2_	33.2, CH_2_	173.3, C	71.0, CH
11	39.3, CH	32.4, CH	-	77.6, C
12	177.3, C	69.2, C	-	26.4, CH_3_
13	17.5, CH_3_	17.0, CH_3_	-	25.1, CH_3_
14	18.1, CH_3_	18.1, CH_3_	25.5, CH_3_	31.4, CH_3_
15	65.2, CH_2_	65.2, CH_2_	65.2, CH_2_	65.1, CH_2_
OCH_3_	51.8, CH_3_	-	52.3, CH_3_	
OAc	-	171.4, C	-	
	-	21.1, CH_3_	-	

**Figure 3:**
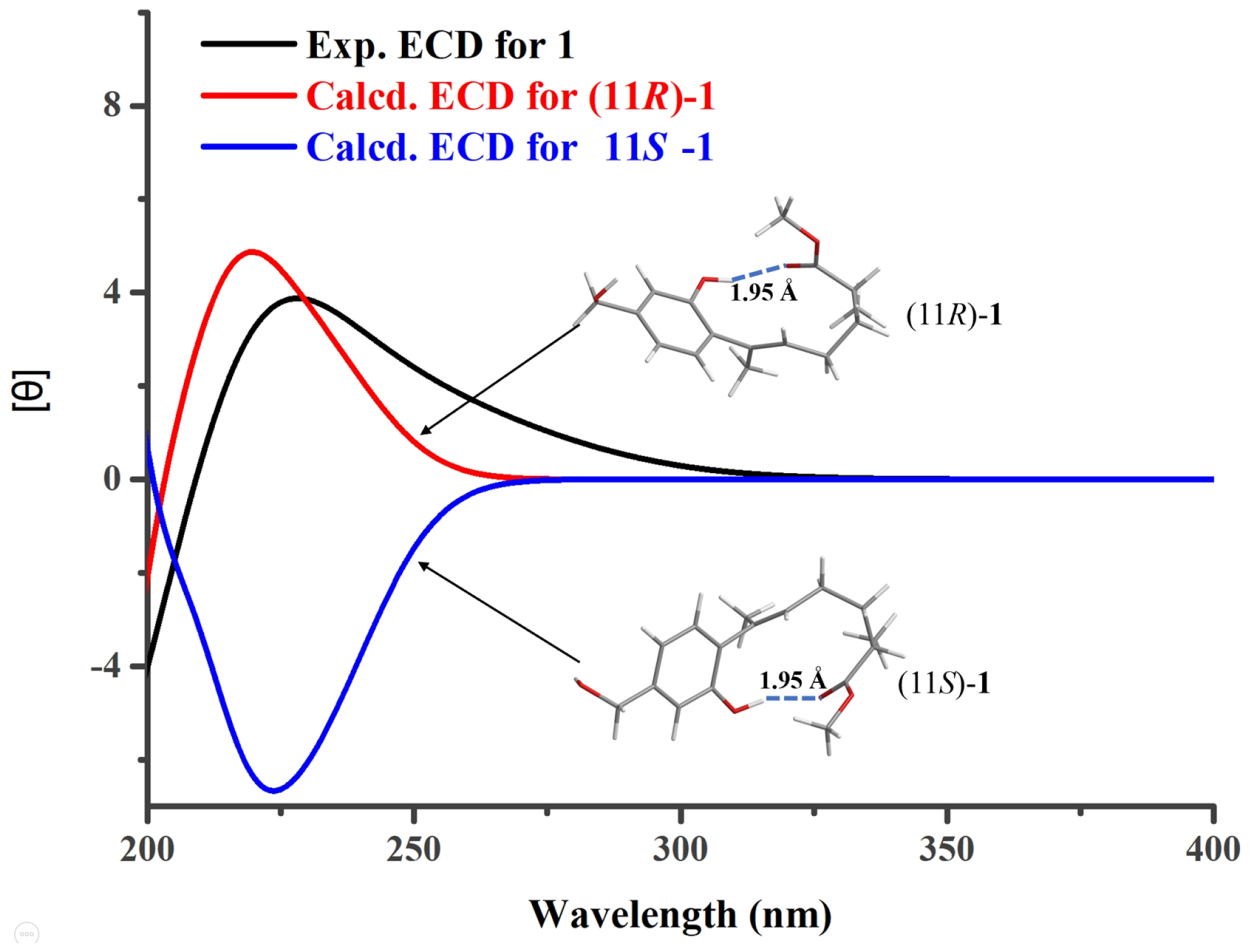
Experimental and calculated ECD curves for **1**.

The determination of the absolute configuration of **1** posed a considerable challenge. First, due to the remote location of the chiral center C-11 from the chromophore in **1**, the ECD cotton effect may not be pronounced enough to enable the use of computed ECD methods for its absolute configuration identification. Second, the low experimental optical rotation (OR) value (−8.4) of **1** also limited the application of determining its absolute configuration through comparison of OR values. However, during the testing of compound **1**’s ECD spectrum, it was unexpectedly observed that a strong cotton absorption peak was present in its experimental ECD spectrum. Subsequently, two possible configurations of **1**, namely, (11*R*)-**1** and (11*S*)-**1**, were used to calculate their ECD spectra. The results indicated that the ECD calculated spectrum of (11*R*)-**1** agreed well with the experimental spectrum of **1** (Figure 3), suggesting that the absolute configuration of **1** was 11*R*. Further analysis revealed that the cause of the ECD cotton effect in **1** lay in the hydrogen bond formed between the 1-OH group and the methoxy group in its 3D conformation. This hydrogen bond, with a length of 1.95 Å and a strong force, enabled **1** to adopt a structure resembling that of resorcylic acid lactones (Kuttikrishnan et al., 2022) in solvents, thereby producing the ECD cotton effect. This discovery reminds us that in evaluating whether a compound could produce an ECD cotton effect, it is necessary to conduct a thorough analysis of its 3D conformation, rather than relying solely on planar structural analysis.

**Figure 4:**
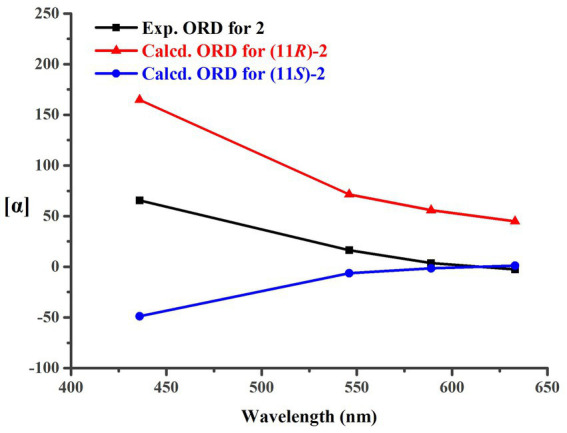
Experimental and calculated ORD curves for **2**.

Trichoderene B (**2**) was also obtained as colorless oil. The similar NMR spectra of **2** (Tables 1, 2) and **8** suggested that **2** should be a bisabolene-type sesquiterpene derivative. Detailed analysis of NMR differences between **2** and **8** indicated that **2** was the result of acetylation of the 12-OH in **8**, which was further verified by the key HMBC correlation from H_2_-12 to -O *C* OCH_3_ (Figure 2). It was also a significant challenge to determine the absolute configuration of **2**. Unlike compound **1**, the 1-OH group in **2** could not form an intramolecular hydrogen bond with the oxygen on the chain, resulting in a weak experimental ECD cotton effect that cannot be applied to its configuration identification. Fortunately, compound **2** displayed a relatively large OR value, which changed with the testing wavelength, forming a well-defined optical rotation dispersion (ORD) spectrum (Figure 4). Based on this characteristic, the calculated ORD spectra of the two possible configurations of **2**, (11*R*)-**2** and (11*S*)-**2**, were applied. The results indicated that the calculated ORD spectrum of (11*R*)-**2** matched well with the experimental spectrum of **2**. Therefore, the absolute configuration of **2** could be confidently determined as 11*R*.

**Figure 5:**
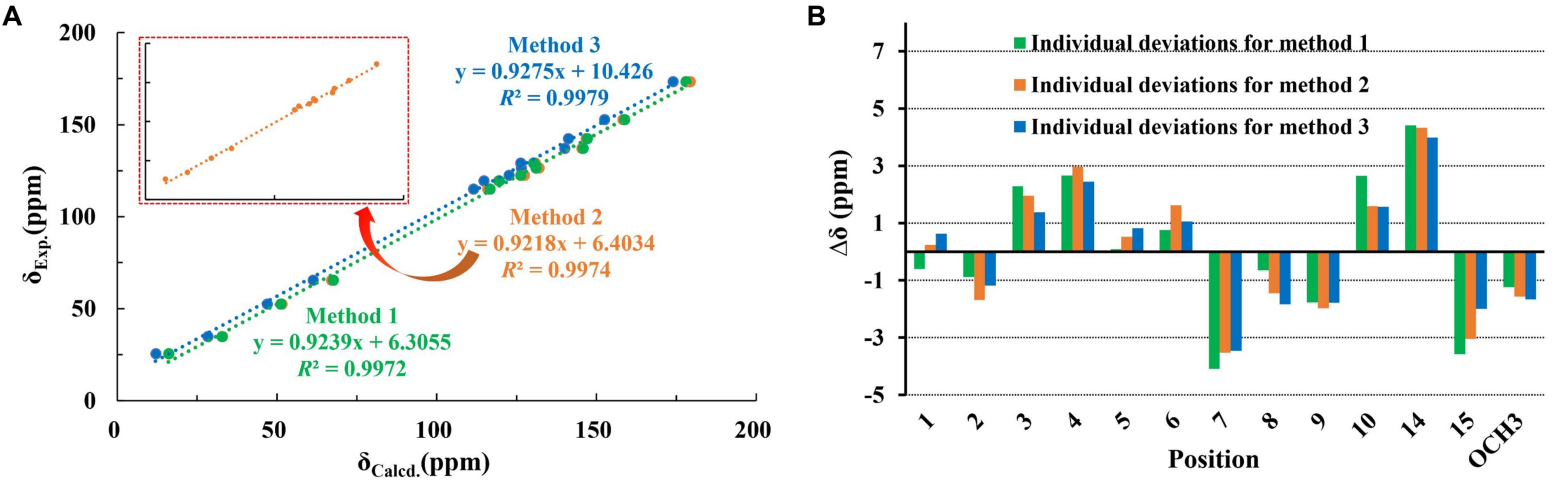
Regression analysis and individual deviations of experimental vs. calculated 13C NMR chemical shifts of **3**.

Trichoderene C (**3**) was also isolated as colorless oil. Although 1D NMR signals (Tables 1, 2) suggested that **3** might belong to the sesquiterpene derivative, its ^13^C NMR spectrum comprised only 13 carbons, including a methoxy carbon, which did not align with the typical 15-carbon skeleton of sesquiterpenes. This indicated that **3** was likely a nor-sesquiterpene. By carefully compared with the reported NMR data of **3** and **1**, it was found that the signals of -CH_2_-CH(CH_3_)-group between C-9 and C-12 in **1** were disappeared in **3**. The key correlations from H-8 and -OC *H* _3_ to C-10 (Figure 2) confirmed the nor-sesquiterpene skeleton of **3**. To validate the skeleton of **3**, three chemical quantitative calculation methods, namely, B3LYP/6–311 + G(d,p) (method 1), B3LYP/6–311 + G(d,p) (PCM, CHCl_3_) (method 2), and mPW1PW91/6–311 + G(d,p) (PCM, CHCl_3_) (method 3), were employed to compute the ^13^C NMR data of **3**, and the computed results were compared with experimental values. The findings revealed that under all three methods, the calculated ^13^C NMR data exhibited good fits with the experimental values, with high correlation coefficient *R*^2^ values of 0.9972, 0.9974, and 0.9979, respectively (Figure 5A). In addition, the maximum error between the calculated and experimental ^13^C NMR data did not exceed 4.4 ppm for any of the three methods (Figure 5B). Thus, the carbon skeleton of **3** was definitely assigned and verified.

**Figure 6:**
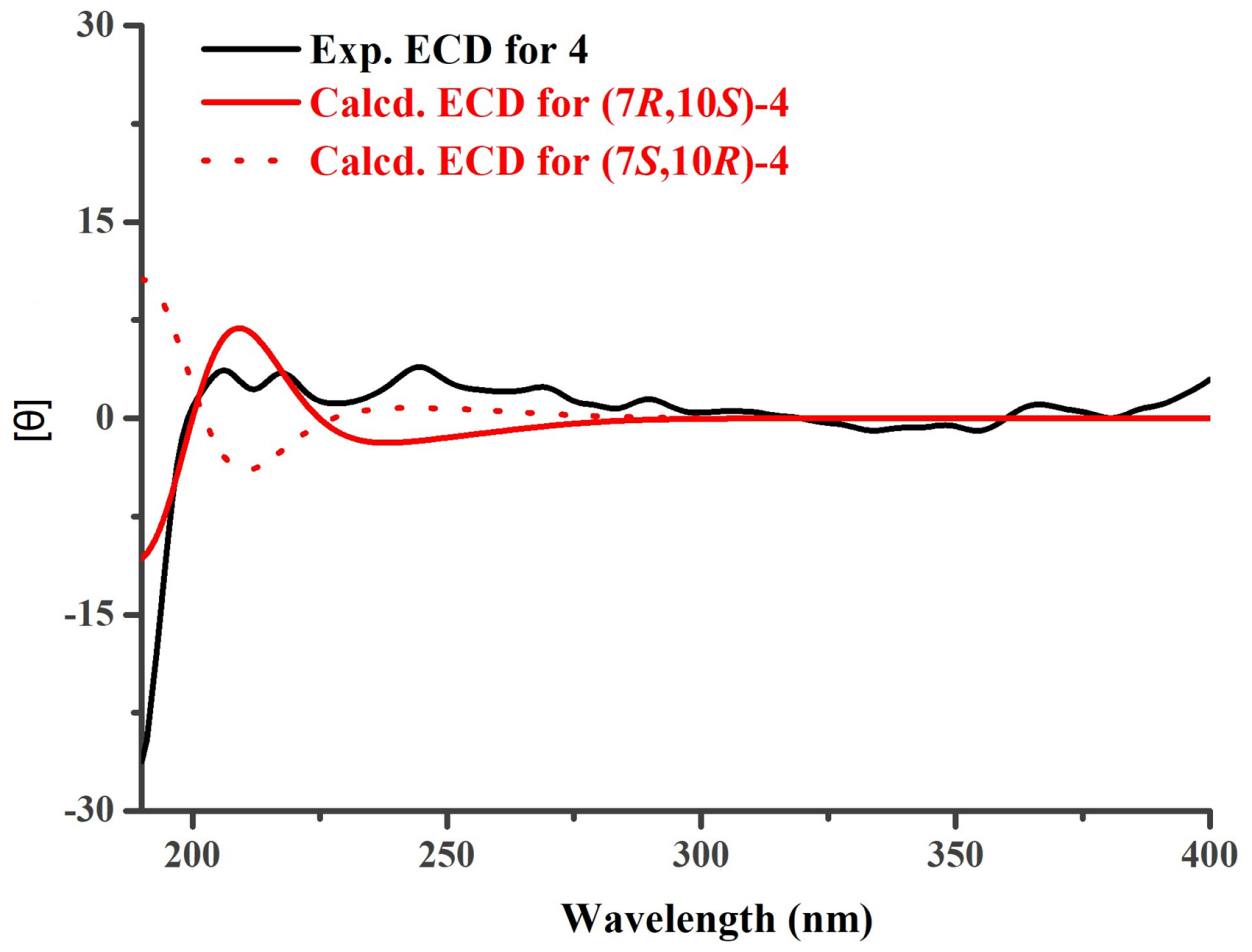
Experimental and calculated ECD curves for **4**.

Trichoderene D (**4**) was also isolated and identified as sesquiterpene analogs according to its NMR data (Tables 1, 2). Its molecular formula was determined as C_15_H_22_O_4_ based on its HRESIMS data, suggesting five degrees of unsaturation. In **4**, the benzene ring accounted for four degrees of unsaturation, thus requiring the side chain to form an additional ring to occupy the fifth degree of unsaturation. In fact, the structure of **4** was as analogous to the known compound **7**, with the main difference being the presence of an additional hydroxyl group at C-10 in **4**. This inference could be confirmed by the key HMBC correlations from H_2_-8 and H_3_-13 to C-10, and ^1^H–^1^H COSY correlation of H_2_-8/H_2_-9/H-10 (Figure 2). In the NOESY experiment, the correlations between H_3_-13 and H-10, and H_3_-12 and H_3_-14 suggested that H-10 and H_3_-14 were located on opposite sides of the molecular. To accurately determine the absolute configuration of **4**, ECD chemical quantitative calculations were performed on two possible configurations of **4**, (7*R*,10*S*)-**4** and (7*S*,10*R*)-**4**. The results indicated that the calculated ECD spectrum of (7*R*,10*S*)-**4** matched well with the experimental ECD spectrum of **4** (Figure 6), suggesting that the absolute configuration of **4** was 7*R*,10*S*.

The known compounds **5**–**10** were identified as cyclowaraterpol A (**5**) (Henne et al., 1993), cyclowaraterpol B (**6**) (Henne et al., 1993), (*S*)-(–)-5-(hydroxymethyl)-2-(2,6,6-trimethyltetrahydro-2*H*-pyran-2-yl)phenol (**7**) (Wang et al., 2016), waruterpol (**8**) (Henne et al., 1993), anhydrowuruterpol A (**9**) (Henne et al., 1993), and (7*S*,11*S*)-(+)-11-hydroxyl-sydonol (**10**) (Ye et al., 2019), by comparing their NMR data with the reference data.

**Figure 7:**
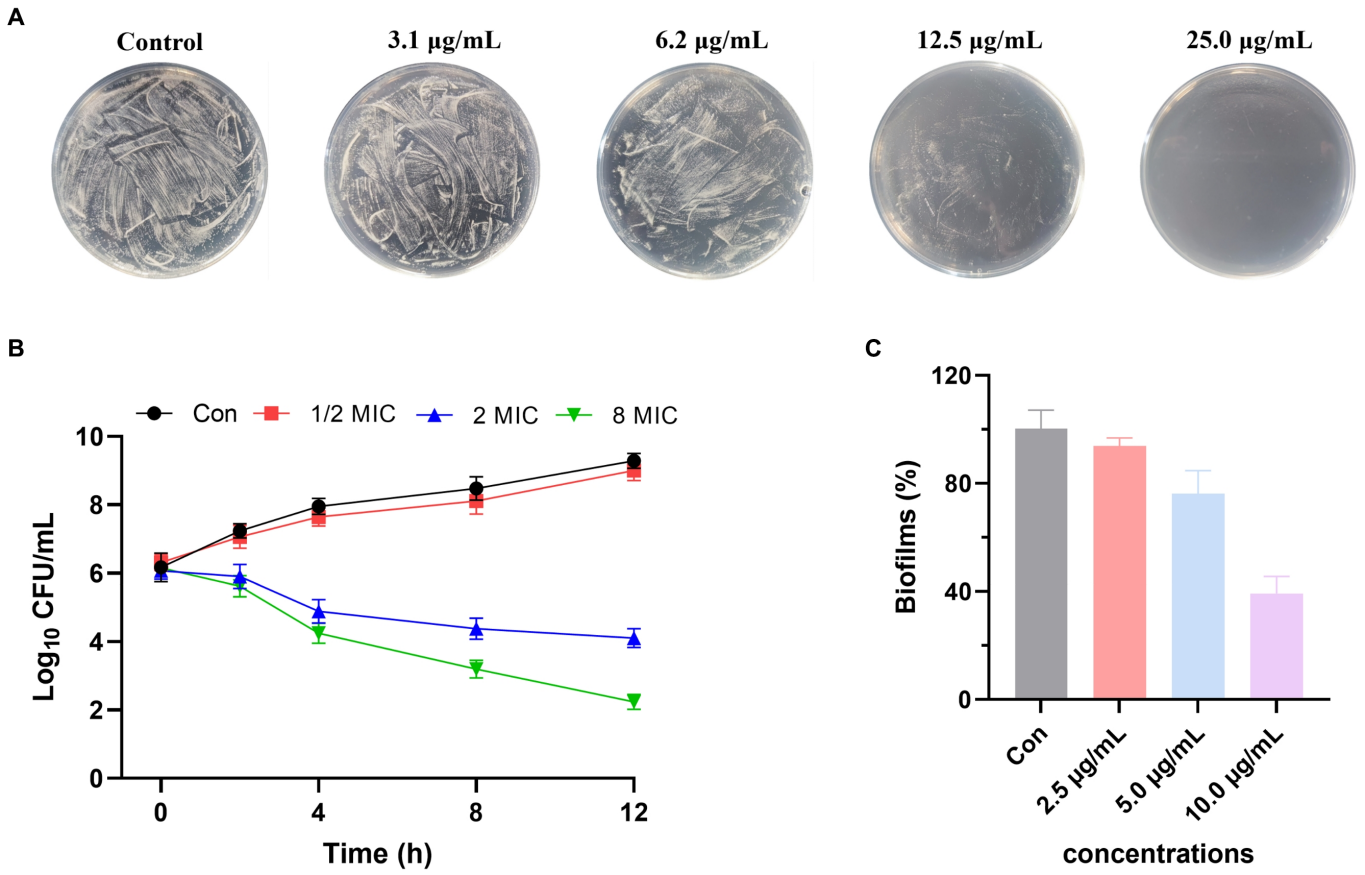
**(A)** Anti-*Agrobacterium tumefactions* activity of **1** using plate spread inhibition assay. **(B)** Time-kill curves of **1** against *A. tumefactions*. **(C)** Biofilm inhibition activity of **1** against *A. tumefactions*.

Anti-*A. tumefactions* activity of the isolated compounds **1**–**10** was then determined. In the conventional 96-well broth dilution assay, compounds **1**–**3** and **8**–**10** exhibited inhibitory activity against *A. tumefactions* with MIC values of 3.1, 12.5, 12.5, 6.2, 25.0, and 12.5 μg/mL, respectively. However, compounds **4**–**7** did not inhibit *A. tumefactions* (MICs >25.0 μg/mL). This indicated that the formation of a cyclic structure in the side chain of these compounds could reduce their anti-*A. tumefactions* activity. To further confirm the antibacterial activity of these compounds, a plate spread inhibition assay was conducted on **1**. The results showed that at a concentration of 6.2 μg/mL, compound **1** inhibited the growth of *A. tumefactions* on the plate. When the concentration of **1** reached 25.0 μg/mL, the growth of *A. tumefactions* on the plate was completely inhibited (Figure 7A). Subsequently, the bactericidal time-kill curve was conducted for **1**, testing its effect at various concentrations including blank, 1/2 MIC, 2 MIC, and 8 MIC (Figure 7B). The results indicated that when the concentration of **1** reached 2 MIC and 8 MIC, bacterial killing began to manifest within 2 h. Notably, at 8 MIC concentration, nearly all bacteria were eradicated within 12 h. Furthermore, the impact of **1** on the formation of bacterial biofilm by *A. tumefaciens* was investigated (Figure 7C). It was revealed that **1** exhibited certain anti-biofilm activity. At a concentration of 5.0 μg/mL, the formation of bacterial biofilm was moderately inhibited, whereas at 10.0 μg/mL, the inhibition was highly pronounced.

## 3 Materials and methods

### 3.1 General experimental procedures

The general experimental procedures were basically consistent with our previous literature (Cao et al., 2023).

### 3.2 Fungal materials

The marine-derived fungal *Trichoderma effusum* HBU-2019-190, originating from the Bohai Sea, was identified and subsequently registered in the NCBI GenBank under accession number MN644788. The fungal strains have been deposited in the collection of the College of Life Sciences, Hebei University, China. *Agrobacterium tumefaciens*, originally separated from soil, was sourced from China Center of Industrial Culture Collection.

### 3.3 Fermentation, extraction, and isolation

In 1,000 mL Erlenmeyer flasks, the fungus HBU-2019-190 was fermented using rice solid medium, with a total of 100 flasks fermented, each containing 100 g of rice and 100 mL of water. The fermentation conditions were set at 28°C for 28 days. After fermentation, a 1:1 mixture of MeOH/CH_2_Cl_2_ was used to extract the fungus for six times. The extract was then dried using a rotary evaporator, resulting in 402 g of crude extract. Subsequently, the crude extract was further extracted with EtOAc and H_2_O to obtain 213 g of EtOAc extract. The obtained EtOAc extract was then subjected to vacuum column chromatography using a petroleum ether (PE)/EtOAc gradient system. The gradient system was set as 90% PE, 60% PE, 30% PE, and 100% EtOAc, resulting in four fractions Fr.1–Fr.4. Among them, Fr.2 was further purified via silica gel column chromatography with a mixture of PE/EtOAc (1:1) as the mobile phase, resulting in four subfractions, Fr.2.1–Fr.2.4. Then, Fr.2.2 was further separated by reversed-phase silica gel chromatography with 80% MeOH as the mobile phase, followed by semipreparative HPLC (MeOH:H_2_O = 40:60, 2.0 mL/min), ultimately yielding compounds **1** (32.0 mg), **2** (24.0 mg), **4** (16.0 mg), **5** (4.3 mg), **6** (4.6 mg), and **7** (1.5 mg). Fr.3 was separated by Sephadex LH-20 chromatography using a mixed solvent of PE, MeOH, and CH_2_Cl_2_ in a ratio of 2:1:1 as the mobile phase, resulting in five subfractions, Fr.3.1–Fr.3.5. Among them, Fr.3.3 was further purified through silica gel column chromatography and HPLC preparation, leading to the isolation of compounds **3** (12.0 mg), **8** (9.5 mg), **9** (2.2 mg), and **10** (3.7 mg).

Trichoderene A (**1**): Pale yellow oil; [*α*]25 D = −8.4 (*c* 1.00, MeOH); UV (MeOH), *λ*max (log *ε*) 246 (4.20), 305 (2.83) nm; ECD (5.2 *μ*M, MeOH), *λ*max (Δ*ε*) 228 (3.87) nm; ^1^H and ^13^C NMR data (see Tables 1, 2); HRESIMS *m/z* 301.1404 [M + Na]^+^ (calcd. for C_16_H_22_NaO_4_^+^ 301.1410).
Trichoderene B (**2**): Pale yellow oil; [*α*]25 D = −70.9 (*c* 1.00, MeOH); UV (MeOH), *λ*max (log *ε*) 245 (4.25), 304 (2.81) nm; ^1^H and ^13^C NMR data (see Tables 1, 2); HRESIMS *m/z* 315.1563 [M + Na]^+^ (calcd. for C_17_H_24_NaO_4_^+^ 315.1567).
Trichoderene C **(3)**: Pale yellow oil; UV (MeOH), *λ*max (log *ε*) 244 (4.61), 307 (2.86) nm; ^1^H and ^13^C NMR data (see Tables 1, 2); HRESIMS *m/z* 259.0938 [M + Na]^+^ (calcd. for C_13_H_16_NaO_4_^+^ 259.0941).
Trichoderene D (**4**): Pale yellow oil; [*α*]25 D = −10.7 (*c* 1.00, MeOH); UV (MeOH), *λ*max (log *ε*) 242 (4.39), 302 (2.74) nm; ECD (5.0 *μ*M, MeOH), *λ*max (Δ*ε*) 206 (3.68), 218 (3.44) nm; ^1^H and ^13^C NMR data (see Tables 1, 2); HRESIMS *m/z* 289.1401 [M + Na]^+^ (calcd. for C_15_H_22_NaO_4_^+^ 289.1410).

### 3.4 Computational section

The different configurational molecules of **1**–**4**, including (11*R*)-**1**, (11*S*)-**1**, (11*R*)-**2**, (11*S*)-**2**, **3**, (7*R*,10*S*)-**4**, and (7*S*,10*R*)-**4**, seven molecules in total, were used for quantitative chemical calculations. Initially, minimum energy conformation search for these molecules was conducted using the Compute VOA software, with relative energy within a 10.0 kcal/mol energy window and the MMFF94 force field applied. This resulted in 47 stable conformers for (11*R*)-**1**, 36 stable conformers for (11*S*)-**1**, 30 stable conformers for (11*R*)-**2**, 36 stable conformers for (11*S*)-**2**, 53 stable conformers for **3**, 12 stable conformers for (7*R*,10*S*)-**4**, and 12 stable conformers for (7*S*,10*R*)-**4**, respectively. Subsequently, these minimum energy conformations were optimized for the first time using Gaussian software at the B3LYP/6-31G(d) level (gas phase). Following the initial optimization, the conformations were ranked based on their energies, and those with an energy difference within 2.5 kcal/mol were selected for a second round of optimization at the B3LYP/6–311 + G(d) level (gas phase). After the second optimization, ECD or NMR calculations were performed on the optimized conformations. Finally, based on Boltzmann statistics, the final ECD and NMR spectra for each configurational molecule were computed.

### 3.5 Anti-*Agrobacterium tumefactions* activity assay

Using 96-well broth dilution assay method (Schug et al., 2020), the anti-*A. tumefactions* activity of compounds **1**–**10** was determined, with ampicillin serving as the positive control, having a MIC value of 0.3 μg/mL. Subsequently, the inhibitory effect of **1** against *A. tumefactions* was evaluated using the plate spreading method (Lewis and Fleming, 1995). Specifically, 20 mL of LB medium containing various concentrations of **1** was poured into a 9 cm-diameter petri dish. The bacteria of *A. tumefactions*, cultured 1 day prior, were then uniformly spread on the plate. Following this, the plate was inverted and incubated at 28°C for 12 h. Finally, photographs were taken to record the growth of colonies. The design of the bactericidal time-kill curve test experiment and bacterial biofilm experiment for **1** was based on previous literature. For time kill assays, tubes were prepared containing freshly prepared LB broth supplemented with compound **1** at various concentrations, including a blank control, 1/2 MIC, 2 MIC, and 8 MIC, along with *A. tumefactions* isolates at a concentration of 10^4^ CFU/mL. The tubes were incubated at 28°C in a shaking incubator (200 rpm). Then, 100 μL aliquots were obtained from each tube at 0, 2, 4, 8, and 12 h of incubation and serially diluted in saline for the determination of viable counts. Diluted samples (10 μL) were plated on LB plates and incubated at 28°C for 12 h, and then, the number of colonies was counted. The lower limit of detection for the colony counts was 2 log_10_ CFU/mL (Foerster et al., 2016). For bacterial biofilm experiment, after overnight cultivation, 100 μL/well of the bacterial culture, diluted in LB broth with 0.5% glucose, was aliquoted into 96-well microplates with 1 μL of different concentrations of **1** and incubated at 37°C for 24 h. After incubation, each well was rinsed with 1 × PBS to remove non-adherent cells and then dried at 37°C. CV staining was used to determine the remaining total biofilm biomass, and the absorbance was measured at 550 nm (Song et al., 2021).

## 4 Conclusion

In conclusion, 10 sesquiterpene derivatives (**1**–**10**), including four new compounds (**1**–**4**), were obtained from the marine-derived fungal strain *Trichoderma effusum* HBU-2019-190 by using bioassay and HPLC guided methods. The chemical structures of these compounds were determined and confirmed through extensive spectroscopic methods and chemical calculations. Notably, some of these compounds exhibited strong inhibitory activity against *Agrobacterium tumefaciens*, providing significant value for the development of novel anti-*A. tumefactions* pesticides.

## Data availability statement

The original contributions presented in the study are included in the article/supplementary material; further inquiries can be directed to the corresponding authors.

## Author contributions

YL: Methodology, Writing – original draft. LQ: Data curation, Formal analysis, Writing – original draft. MX: Formal analysis, Investigation, Writing – original draft. WL: Data curation, Methodology, Writing – original draft. NL: Supervision, Writing – review & editing. XH: Funding acquisition, Supervision, Writing – review & editing. YZ: Funding acquisition, Supervision, Writing – review & editing.
